# Hallucinations and Delusions as Low-Quality Attributions: Influencing Factors and Proposal for Their Analysis

**DOI:** 10.3389/fpsyg.2021.533795

**Published:** 2021-07-23

**Authors:** Juan F. Rodríguez-Testal, Cristina Senín-Calderón, Rafael Moreno

**Affiliations:** ^1^Personality, Evaluation and Psychological Treatment Department, University of Seville, Seville, Spain; ^2^Department of Psychology, University of Cádiz, Puerto Real, Spain; ^3^Department of Experimental Psychology, University of Seville, Seville, Spain

**Keywords:** delusions, hallucinations, attributions, field model, psychosis

## Abstract

Hallucinations and delusions, in keeping with the distress accompanying them, are major features in the diagnosis of psychosis in international classifications. In spite of their human and clinical importance, the concepts are unclear. The distinction between hallucinations and delusions in terms of perception-thought is not precise enough, causing problems in analyzing the patient’s words. Nor are the differentiations or variations within each precise enough. Continuing the long clinical tradition discussing the distinction between hallucinations and delusions while assuming their similarities, this study poses a concept integrating the two phenomena as attributions people make about themselves and their settings. Then the elements of any attribution can be used as guides for structuring significant literature on both, and reduce analytical ambiguity. Such attributions make more sense within the structure of two-way relationships with factors in a person’s own framework and setting. This structure is described with its variables and relationships as a guide to assessment, follow-up, and intervention. Two checklists are provided for orientation.

## Introduction

Hallucinations and delusions are manifestations that cause those who experience them enormous suffering (Varese et al., 2016; Garety et al., 2020). They are major factors in the diagnosis of psychotic disorders in the most widely used classifications (American Psychiatric Association [APA], 2013; World Health Organization [WHO], 2018). Hallucinations are Esquirol’s classical “perception without object” (1817), and delusions are basically thoughts which do not fit reality (Jaspers, 1913).

In spite of their human and clinical significance, the concepts are problematic. The definitions in the literature are themselves unclear (Garety et al., 2020) and varied (Sheaves et al., 2020) leading to oversimplification. For example, a patient says he hears the voice of God. Although the reference to what he hears seems clear, it is not so clear why he says it is the voice of God, or what about that voice makes him identify it as such. Their distinction in terms of perception-thought is imprecise, making analysis of the patient’s words hard to examine. As discussed further below, it is often not clear if they really hear or see what they say they hear or see, or for example, as one woman said, “They talk and talk, but not like you or me. It’s different.” In this sense, many hallucinatory phenomena, such as orders and comments, can be considered intrusive thoughts (McCarthy-Jones et al., 2014), and the intrusive thoughts of patients with obsessive-compulsive disorder may have audible or visible characteristics (Moritz et al., 2018). Voices have both sound and thought (Woods et al., 2015), and hallucinations may have sensory elements related to thought (Jones and Luhrmann, 2016). For example, one patient we assessed said, “I realize I think those things…, but no, I don’t, it’s the voices that are telling me that, and they are very unpleasant.” This example includes another problem, because what this person at first identified as a thought, he later denies, or confuses with the hallucinations he experiences. Furthermore, around 10% of auditory hallucinations are inaudible (Moritz and Larøi, 2008), and some patients explain their hallucinations as voices heard with the mind more than with their ears (Henriksen et al., 2016).

Differentiations made within each phenomenon are also imprecise. Although some hallucinations may be identified as auditory, tactile, or visual, others are not described, such as “The speaker on TV is talking to me in a special way,” “I don’t know if I saw it or not, but it was there,” “A lot of people are saying bad things about me, that I should kill myself and that, but I don’t understand what they are saying, they don’t say it in words.” This may suggest that such sensory types are culturally molded expressions (Laroi et al., 2014). Delusions, in the phenomenological tradition, are separated into primary, that is unintelligible, or true delusions, and secondary delusions, those understandable as life experiences (like pathological jealousy or most of the persecutory delusions) (World Health Organization [WHO], 2018). Nevertheless, this distinction of secondary delusions as the patient’s effort at interpretation (Maher, 2006) is unnecessary, because of the difficulty in explaining an experience with no clear empirical referent in primary delusions (Cermolacce et al., 2010). For example, “I feel like I’m rotting,” “I feel like I don’t have a soul anymore,” or “I don’t have a body.” And, however, in this same line of research, the importance of the experiential or live nature of the delusion is emphasized (Feyaerts et al., 2021). That problem appears again in differentiating between autistic delusions, considered metaphors of the subjective experience, and therefore, similar to primary delusions, and empirical delusions (Parnas et al., 2010), which have a realistic referent. Other studies have relativized both the distinction between the two phenomena, and within them (Dudley et al., 2018; Maijer et al., 2018).

Continuing the discussion of the problems already mentioned above, a long clinical tradition argues for the hallucination-delusion distinction. Séglas (1982) referred to hallucinations as thoughts audible only to the person himself. Building on the works of Jaspers (1913), hallucinations have been described as objectification of inner speech, spatialization of experience, or perception of consciousness (Sass and Parnas, 2003), as a cognitive phenomenon (Handest et al., 2016) not exclusively perceptive-sensory. This is the case of a patient who said she recognized her husband’s mistress, was completely convinced that she had finally found out who she was, and that she had seen her, and at the same time, mentioned that it was raining at the time and she could not see her face because of the umbrella, and all the while insisted she did not know who the woman was. Another patient was not sure if he knew or saw two policemen in a helicopter, saying they were smiling because they were going to trap him (while he was on a bus). The doubts about what patients hear are found in Bleuler (1960), and in Clérambault (1926) who described mental automatism as beginning without sensory reference. So, the problem in distinguishing between thought and perception may come from analyzing and later elaborating on cognitive phenomena. This again makes perception-thought an oversimplified and imprecise distinction. Alternatively, hallucinations and delusions have been considered on a continuum (Strauss, 2014) between two extremes related to the attenuated sense of self (Rosen et al., 2016), or similar processes (Bentall, 1990), as a single dimension distorting reality (Barch et al., 2013) and extensive to any positive symptom (Moritz et al., 2017). The first objective of this study was therefore to contribute a concept with parameters that can integrate the findings recognized in the literature as hallucinations and delusions, enabling both to be understood as different cases of that single concept.

Nevertheless, some models have emphasized factors that are significant in the disorders discussed here. Some are about the person. Cognitive models have underlined the importance that unfulfilled basic human needs, such as affiliation, security, or care have on altered functioning (Gilbert, 2001; Campbell and Morrison, 2012), development of one’s own schemas (about the self, the world around us, and the future) (Beck et al., 2019), avoidance or escape behaviors, or interpretations of the symptoms themselves (Birchwood et al., 2000; Tully et al., 2017). Family therapy models suggest that deficient communication, coping, and self-control are important to symptoms (Palazzoli et al., 1977; Liotti and Gumley, 2009). Other factors refer to the setting. Finnish intervention in psychosis suggests the importance of conditions favoring recovery, such as care and its immediacy, dialogue, trust, and tolerance to uncertainty by the patient’s support networks (Seikkula et al., 2006; Lakeman, 2014), and lack of family warmth as the best predictor of relapse (Bucci et al., 2016). Other models emphasize factors related to highly stressful situations, such as mourning or abuse (Varese et al., 2012) or chronic stress, social isolation, and social inequality (Read et al., 2013; Bentall et al., 2014). Such factors alter the patient’s emotional state and thoughts about their self-worth (Garety and Freeman, 2013), leading to hallucinations and delusions in response to chronic, highly intense stress (Geekie et al., 2012; Pienkos et al., 2019). Current phenomenological perspectives also consider factors related to personal experience, such as loss of familiarity and continuity in inner experiences linked to identity. This affects loss of automatic cognitive, motor, and emotional processes, involving loss of agency, experience of vitality, integration, and sense of belonging (Henriksen and Parnas, 2017). Loss of continuity or speed of thought favors voicing of thought, personification, or spatialization of inner speech (Parnas and Zandersen, 2018). Given the significance of these factors in hallucinations and delusions, they should be considered in the integrating concept sought. Thus, the second objective of this study was to construct a model that could achieve this.

Moreover, both concept and model had to be delimited precisely and clearly differentiated from each other, assembling the aspects of the phenomena under study and the factors mentioned in the literature discussed above.

### Integrating Concept Proposed

This study integrates hallucinations and delusions by classifying them both as *attributions* people make about their settings and themselves. Then, the elements of any attribution may be used as guides for analyzing significant literature on both, and reduce the current ambiguity.

**FIGURE 1:**
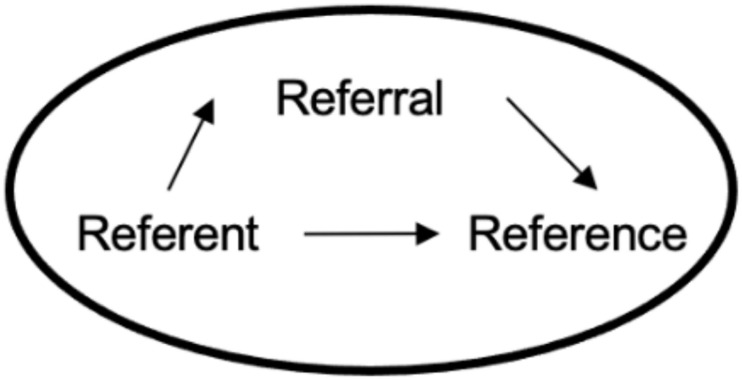
Structure of the components of any attribution.

*Attribution* is therefore defined here as a person’s interpretation of content or meaning assigned to someone or something, and involves the following elements: *Reference*, the content or meaning assigned; *referral*, the person or thing the content of the reference is assigned to, and *referent*, the agent or generator of the reference. Thus, the reference is the product of the referent with mediation of the referral (see Figure 1). This analysis was developed based on the hypotheses of Wilkinson and Bell (2016) on agency in hallucinations and delusions.

Each attribution element includes several parameters with different cases or possibilities, as summarized in Table 1. *Location* is a parameter for both the referent and the referral. The referent may be the person who makes the attribution, as in “I am dead,” but could also be someone else, or something that makes the attribution, as in “God hates me” or “Those antennas are watching me.” The same is true of the referral, as in “They’re watching me” or “They’re watching my father,” respectively, and those others could be located within the person, as in “I have another person moving inside my body.” There are two groups of reference parameters. The first is the content of the attribution and includes two parameters. One is the *theme*, with possibilities mentioned in the literature: paranoid, passionate, somatic, etc. Another parameter is the *purpose* identifiable in the content. These could be advice, compliments, and so forth, grouped in three types: Imposition (e.g., “It was when I looked him in the eye that I knew he was going to kill me”), suggestion (e.g., “I was really annoyed because they said I should buy a lottery ticket, but I didn’t want to, because I don’t have any money.”), or neutral (e.g., “I don’t care, I don’t pay any attention to them,” when the voices say they are going to kill him). The second group of parameters, composition, includes *structure and modality*. Structure may be relational if the attribution consists of some reasoning which connects ideas, and non-relational if it consists of a mere cluster of ideas. Examples of each are, “They want to kill me because I am a spy” and “I am a spy, the Pope, and I like to eat with my fingers.” In modality, the possibilities consist of expressing the attribution reference as a thought or as sensory perception, as in delusions and hallucinations, respectively, or a combination of the two, as well as the ambiguity mentioned in the literature. An example of the combination of attributions, with or without sensory reference, would be one of the more common clinical expressions. For instance, a patient who says he has been worried for a long time because he has stopped helping his father in his bar, and that his father had gotten so angry that he was harassing him (which is not what his family says); that he started to hear the neighbors criticizing him, and later, how he clearly sees how he is shot in the head, from which he deduces that Basque terrorists are behind all his father’s insistence.

**TABLE 1:** Parameters of the elements of attributions.

**Attribution element**	**Parameter group**	**Parameters**	**Cases or possibilities**
Referent		Location	The agent or generator of the reference
			A person or object other than the person who attributes
			Another person or object, within the person who attributes
Referral		Location	The person or thing the content of the reference is assigned to
			Another person or object outside of the person who attributes
			Another person or object within the person who attributes
Reference	Content	Theme	Paranoid (persecution, harm, hurt, referential)
			Maniacal (grandeur or megalomania)
			Passionate (jealousy, infidelity, Clérambault)
			Somatic (illness, nihilism, parasitosis)
			Depressed (guilt, ruin)
			Other
			Anideic
		Purpose	Imposition
			Suggestion
			Neutral
	Composition	Structure	Relational, more or less complex
			Non-relational, with one or several contents
		Modality	Not perceived with the senses
			Involving sensory perception
			Both of the above
			Indefinite
All three	Expression	Language	Oral
			Written or graphic
			Gestures
	Quality of meaning & form	Precision	Precise, clear
			Ambiguous, imprecise
		Differentiation	No repetitions or partial or complete overlapping
			Repetitions or partial or complete overlapping
		Fit	Fitted
			Biased by excess
			Biased by defect
			Biased by excess and defect

Two last groups of parameters refer to the attribution as a whole. One is the oral, written, and body *language* used to express it. The other is the quality of its meaning and expression. Its parameters include the criteria of scientific validity (Martínez and Moreno, 2014). The first is *precision* or clarity. Some attributions are imprecise in meaning, but precise in how they are expressed, usually known as formal alterations of thought, but with understandable words and syntax, such as “Turning backward first is intelligent and keeps another crazy person with criminal instincts from eating”; “How is it my fault that I have six senses and I was born with a heart and you were not? The drunkard understands me, I am crazy, I am the Supreme Judge, I, Honoris Rex.” However, attributions that could be called a “word salad” would be imprecise in both their meaning and how they are expressed. The second quality parameter is *differentiation*, what makes each attribution different from others, as one patient says: “I really thought I was going to get married when the bells rang, I thought it was like a prize for me (I was in a public square and I heard church bells),” and then she thought they were recording all her telephone conversations. On the contrary, the following example shows overlapping or repeating, and therefore, lack of differentiation, “Then they’re coming to see me?. (…), Are they coming to see me?” And later, “Tomorrow they’re coming to see me?” This does not refer to expressions that include formal alterations, such as perseveration or palilalia. The third parameter of quality is *fit* or how well each attribution corresponds to the external criteria that evaluate it, its different parts, their sequence, and the expression as a whole. For example: “I felt really terrible because a man driving a car insulted me with the letters BCH” (which for her meant bitch), where formal linguistic expression is not the problem, but the attribution of the illogical meaning is based on external parameters. Thus, failure to fit is sometimes due to lack of or insufficient meaning, as in alogia and in expression with telegraphic-type speech, such as “Sick help, …call father,” understandable only in certain conversations. Other failures are from excessive meaning, as in derailing and logorrhea. Other attributions fail to fit due to absence or excess, such as those that are tangential and can be qualified as incoherent or illogical.

### Attribution Framework Factors

The usefulness of attributions increases when their two-way relationships with factors that influence them or are modified by them are considered. That combination of attributions and their framework factors is described here as a field model. This type of model, suggested in various branches of physics, health (Laframboise, 1973; Lalonde, 1974), and psychology (Lewin, 1936; Kantor and Smith, 1975; Ribes, 2018) interprets its subject matter as an inseparable structure of variables and relationships that make mutual sense. The model proposed here is the result of reiterative fitting to a large sample of literature, only partly referenced due to space limitations.

The many possible framework factors of an attribution are grouped below (Table 2) *by person* and *setting*.

**TABLE 2:** Attribution framework factors.

**Factor groups**	**Factors**	**Cases or values**
In the person	Emotions, behaviors, and thoughts	Those in each factor in this group
	Personal needs	Physiological
		Security
		Affiliation
		Esteem or recognition
		Self-realization
In the setting	Family	Those in each factor in this group
	Work	Those in each factor in this group
	Leisure and friends	Those in each factor in this group
	Other	Those in each factor in this group

By person, *emotions* or feelings, *behaviors* or activities, and *thoughts* or ideas are included, either as such, or indirectly and implicitly in variables such as age, gender, or education. Another significant factor is *personal needs*, or a mere threat to them: physiological, such as breathing, eating, or drinking, and avoiding pain, and so forth; or psychological, such as security, affiliation, recognition, and self-realization, as described by Maslow (1943) and adapted by Self-Determination Theory (Deci and Ryan, 2000) or by Liotti and Gilbert (2011). Other also numerous factors in the setting may be classified as family, work, leisure, and other areas, which could also include the therapist, when applicable.

### Effects of Attributions on the Framework Factors

These relationships refer to changes in the framework factors by attributions. They may be personal, such as changes in emotions, behaviors, thoughts, or needs. The resulting emotions may be grouped by their *valence* as pleasant, such as joy, pride, or wellbeing (e.g., “I didn’t want to take the pills because then I wouldn’t hear the voice of Jesus Christ telling me things that I liked to hear”), or unpleasant, such as sadness, anxiety, anger, disgust, perplexity, shame, or guilt, although both could be present (e.g., “I hate my father, but I love him so much,”), and an indefinite valence is also possible. The estimated *intensity* of the emotion identified is also important.

Resulting *behaviors* could even be the absence of any at all, such as in freeze, catatonic inhibition, and classic conversion paralysis (e.g., “I stopped moving because they made my brain feel like wood. They were playing with me. And they have no right!”). Others are attempts to neutralize or reduce the attribution or its influence (e.g., “I couldn’t say anything, because then I would really have gone crazy. I didn’t dare think about anything,”) such as flight and fight behavior (Corr, 2013), or self-harm or playing very loud music as incompatible elements. A third type tries to maintain or increase the attribution (e.g., “At first, I was scared to death, but then I liked to listen to them, because they said, ‘You’re such a good person,’ or ‘you are looking great today!”’). All the behaviors could be either intentional or involuntary, and even include sleeping, eating, anesthesia or hypersensitivity without any organic origin, and sexual appetite. Behaviors should also be considered as help-seeking or *autonomous*.

The resulting cognitions are ways individuals have of understanding their own attributions, or even their absence. They are self-judgments, not made by an outside analyst like the rest of the model’s components. There are two groups: Characterization of the attributions and their quality (see Table 3). Characterization includes: *Origin assigned*, in which the attributions may be understood on their own or imposed by someone or something (e.g., “Understood, I’ll shut up,” and the patient clarifies afterward that when the psychologist cleared his throat during the conversation it was an order to keep quiet immediately); *privacy* of the attributions, whether only known to the one who makes them (e.g., “He’s the one (Jesus Christ) who only says things to me”), or by someone else, as in diffusion or thought theft; *controllability*, or the possibility of the attribution somehow being interfered with, thoughts being inserted in their absence (e.g., “It’s the only way,” making a strange repetitive sound, “It’s how they stop bothering me,” referring to the voices); and the *sense* they make of attributions as beneficial, harmful, or neutral (e.g., “Some bricklayers started to work across the way, and then I knew that everything was wrong and I had to throw everything out the window”). The quality or validity they grant their own attributions is considered with the same criteria as the precision, differentiation, and fit applicable by the analysist and summarized in Table 1, but modified to emphasize that they are the individual’s own opinion. *Clarity* granted the meaning of the attribution itself, whether sharp or ambiguous (e.g., “I say it is something like, as if they grabbed you from behind and held you back, but I’m not sure if it’s that or what”); *distinction* from other attributions they have is whether they are distinct or overlap with others; and *credibility of* the attributions themselves, which could be convincing or question whether they are true, whether by outside inference or without it as in saying, “I am the son of the Count of Peñaflor… What a dumb thing I just said!” Personal needs resulting from the attributions, satisfying or reducing, maintaining or increasing them, are all possible. Changes in setting factors, such as the person being treated differently in the family or work environment after the delusions or hallucinations, are also possible.

**TABLE 3:** Effects of the framework factors.

**Effect groups**		**Effects**	**Cases or values**
Emotional		Valences	Pleasant (joy, pride, wellbeing, etc.)
			Unpleasant (sadness, anxiety, anger, disgust, perplexity, shame, guilt, etc.)
			Both valences
			Indefinite
		Intensity of the valence	High
			Low
Behavioral		Behavioral	Absence of behavioral effects (freeze, catatonia, etc.)
			Neutralizing the attribution (flight, fight, etc.)
			Maintaining or increasing the attribution
		Autonomy	No outside help
			Seeking outside help
Cognitive	Characterization of the attribution	Origin assigned	Own
			Elsewhere
		Privacy	Personal or private
			Public or shared
		Controllability granted	Yes, with degrees
			None
		Sense considered	Beneficial to the person
			Harmful
			Neutral
	Quality given the attribution	Clarity granted meaning	Clear
			Ambiguous
		Distinction considered	Distinct
			Overlapping
		Credibility given	Conviction or credibility
			Questioning the attribution by external induction
			Questioned by oneself, without outside inference
Personal needs			Reduction or satisfaction
			Maintenance
			Increase
In setting factors		Family	Changes or not
		Work	Changes or not
		Leisure, friends	Changes or not
		Other	Changes or not

### Influence of the Framework Factors on Attributions

These relationships are changes in the parameters of the attributions as a result of changes in the person’s framework factors or setting. Some influence attributions of needs not met, such as insecurity favoring paranoid themes, lack of recognition favoring manic themes, affiliation favoring passionate themes, need for security, somatic themes or self-realization and affiliation, and depression (Gilbert et al., 2007; Carvalho et al., 2013). An example of the influence of the setting would be invalidating environments (Lungu and Linehan, 2017).

### Relationships Between Framework Factors

**FIGURE 2:**
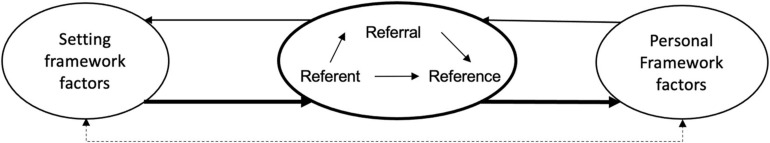
Attributions and their framework factors.

In the relationships discussed above, attributions affect framework factors and these in turn influence the attributions, as shown in Figure 2, with heavy and fine continuous lines, respectively. In addition, the relationships of the framework factors should be considered, whether personal, in the setting, or of one type with the other. When the attributions are not included, they are considered of secondary importance and are shown in Figure 2 as dashed lines.

### Diachronic Perspective

The synchronic or current perspective of the relationships described above is matched with a diachronic or longitudinal perspective. This adds additional information. The unit of analysis is expanded from an attribution to a set or *episode* of attributions, or even a *series of episodes*, with a conventionally identified beginning and end. New parameters of the attributions and framework factors now make sense, mainly: *Frequency* of attributions over a certain time and *intervals* between two in a row, in addition to trends, such as changes or stability of these parameters over time. The *series of reciprocal relationships* formed by successive influences and effects of framework factors on the attributions also enter the analysis. This happens, for example, when the work-climate framework favors an attribution, which then leads to changes in the person’s behavior and interaction with the family. This is represented in Figure 2 by the order of these relationships.

### Checklist for Model Application to Clinical Practice

In addition to conceptualizing hallucinations and delusions, the model can describe an individual with synchronic and diachronic analyses of the variables and relationships, which assists in the assessment, follow-up, and intervention in clinical practice, testing the ecological validity, and therefore, the usefulness of our proposal. Two checklists are provided below as a guide, the first for a cross-sectional description (see Table 4), which is filled in following the parameters and cases summarized in Tables 1–3, 5.

**TABLE 4:** Checklist of the synchronic perspective of an attribution.

**Person studied:**
**Date of attribution:**
**Attribution number or code:**
**CHARACTERIZATION OF THE ATTRIBUTION:**
Who or what is the referent of the attribution?
Who or what is referred to by the attribution?
What type of theme is the reference?
What type of purpose does the reference have?
Does the reference relate to or number the contents?
Does the reference involve sensory perception?
What language is the attribution expressed in?
Does the attribution appear with sufficient precision in form and meaning?
Is the attribution differentiated from other expressions in form and meaning?
Does the attribution fit in form and meaning with the convention considered?
**ENUMERATION OF FRAMEWORK FACTORS**
What emotions, behaviors, and thoughts of the person appear to be significant?
What personal needs are detected?
What factors in the setting appear to be significant?
**EFFECTS OF THE ATTRIBUTION ON THE FRAMEWORK FACTORS**
What emotions are associated with the attribution?
To what degree or intensity do those emotions appear?
What behaviors are considered associated with the attribution?
Do these behaviors help neutralize or maintain the attribution?
Does the person think the attribution is his/her own or someone/something else’s?
Does the person think the attribution is public or private?
Does the person have control over the attribution?
Does the person give a beneficial or harmful sense to the attribution?
Does the person think the attribution is clear?
Is the attribution differentiated from other thoughts?
What credibility does the person give the attribution?
Does the attribution have any effect on the person’s needs? What and on which ones?
Does the attribution have any effect on setting factors? What and on which ones?
**INFLUENCES OF FRAMEWORK FACTORS ON THE ATTRIBUTION**
Does the attribution modify any framework factors? Which ones and how?
Does the person ask for help or act autonomously?
**RELATIONSHIPS BETWEEN FRAMEWORK FACTORS**
Are any relationships between framework factors considered relevant? Why?

**TABLE 5:** Influences of framework factors on attributions.

**Influences**	**Cases or values**
On attributions	Modified
	Not modified

By applying the checklist above to the successive fields analyzed in an episode or series of attributions, sets can be described. A second checklist for this is given below (Table 6).

**TABLE 6:** Checklist for a diachronic perspective of the attributional field.

**Person studied:**
**Start and end dates of the series of episodes analyzed:**
**Number of episodes in the series**
**Start and end dates of the episodes analyzed:**
**ATTRIBUTIONS**
What parameters change in the attributions considered?
How often do the attributions occur in each episode? And in the whole series?
What is the average frequency of attributions per episode in the series?
What is the average interval between attributions in each episode? And in the whole series?
Other measures considered of interest:
**FRAMEWORK FACTORS**
What factors in the person were considered in the episodes?
What personal needs were detected in the episodes?
What factors in the setting could be related to the attribution?
**EFFECTS OF THE ATTRIBUTION ON THE FRAMEWORK FACTORS**
What effects of the attributions change in each episode in the series? How?
**INFLUENCES OF THE FRAMEWORK FACTORS ON THE ATTRIBUTION**
What influences of the framework factors on the attributions change in the episodes? How?
**RELATIONSHIPS BETWEEN FRAMEWORK FACTORS**
Have any of the relationships between framework factors changed? How?
**OTHER INFORMATION**
Any other relevant information?

## Discussion and Conclusions

The objective of this study was to integrate hallucinations and delusions as particular cases of attributions, differentiated by whether or not sensory perception is involved only when it is of interest and their expression allows it. The model proposed synchronically and diachronically delimits the parameters of any attribution, which makes sense in relation to the person’s framework factors and settings, enabling significant studies in the literature to be outlined.

In this approach, the meanings of the attributions that hallucinations and delusions correspond to, or the way they are expressed, are low-quality, because they are not precise enough, are not differentiated enough from each other, nor do they fit to evaluative criteria, and frequently occur in one or more episodes or series of episodes, with short intervals between them. The fewer of those characteristics present in the attributions, the more likely they are to be merely errors in interpretation. Therefore, those characteristics enable attribution quality to be graded, from the most common, which could be considered subthreshold, to those involving the clinical problems dealt with here.

The rest of the parameters and relationships discussed form the qualitative type of any attribution. They may be classified by the location of referents and referral, theme and structure of the references, language, origin, privacy, controllability, and the sense they make to the person, as well as by their effects and influences on the framework factors by specifying those involved in each relationship. This can reveal the formation and development of the attributions, identifying more framework factors for clinical attributions and those that modify these framework factors the most. Thus, due to their mutual relationships with personal and setting factors found in the literature, attributions would be accompanied by emotions of a certain intensity, changes in behavior, and characterizations and credibility granted by the person.

The various components of the concept of attribution and framework factors are explained precisely and are sufficiently differentiated to be understood and used in clinical practice for assessment and follow-up, and more indirectly, for intervention in these important manifestations, with the checklists provided here. We also think that this concept of attribution and of the framework factor structures is faithful to the most relevant literature on hallucinations and delusions.

These qualities should be tested by clinicians using the checklists to detect insufficient precision and thoroughness of the attribution components and framework factors included. It would not be surprising that limitations would then appear that would have to be corrected in the model’s design. We are aware that the number of components could make it hard to use in clinical practice. However, that large number is necessary to reflect as much of the extensive literature as possible.

In brief, the structure provided by the model proposed organizes the field of low-quality attributions at the same time it enables them to be differentiated from others involving less impairment. This suggests a gradation from phenomena that are not necessarily pathological, such as self-references, overvalued ideas, or depersonalization (Reininghaus et al., 2016; Baumeister et al., 2017; Bell and O’Driscoll, 2018), not discussed here for reasons of space. This gradation would enable validation of the study of low-quality attributions under the conceptual umbrella of the extended psychotic phenotype (Van Os and Reininghaus, 2016). It would also help adjust the relationship between the different phenomena under study in determining the onset and maintenance of the psychotic process.

## Author Contributions

JR-T and RM were fully involved at all stages in the writing of the manuscript. CS-C managed the literature searches. All authors contributed to and have approved the final manuscript.

## Conflict of Interest

The authors declare that the research was conducted in the absence of any commercial or financial relationships that could be construed as a potential conflict of interest.

## Publisher’s Note

All claims expressed in this article are solely those of the authors and do not necessarily represent those of their affiliated organizations, or those of the publisher, the editors and the reviewers. Any product that may be evaluated in this article, or claim that may be made by its manufacturer, is not guaranteed or endorsed by the publisher.

## References

[B1] American Psychiatric Association [APA]. (2013). *Diagnostic and Statistical Manual of Mental Disorders (DSM 5).* Arlington, VA: APA.

[B2] Barch D. M. Bustillo J. Gaebel W. Gur R. Heckers S. Malaspina D. (2013). Logic and justification for dimensional assessment of symptoms and related clinical phenomena in psychosis: relevance to DSM-5. *Schizophr. Res.* 150 15–20. 10.1016/j.schres.2013.04.027 23706415

[B3] Baumeister D. Sedgwick O. Howes O. Peters E. (2017). Auditory verbal hallucinations and continuum models of psychosis: a systematic review of the healthy voice-hearer literature. *Clin. Psychol. Rev.* 51 125–141. 10.1016/j.cpr.2016.10.010 27866082 PMC5240854

[B4] Beck A. T. Himelstein R. Grant P. M. (2019). In and out of schizophrenia: activation and deactivation of the negative and positive schemas. *Schizophr. Res.* 203 55–61. 10.1016/j.schres.2017.10.046 29169775

[B5] Bell V. O’Driscoll C. (2018). The network structure of paranoia in the general population. *Soc. Psychiatry Psychiatr. Epidemiol* 53 737–744. 10.1007/s00127-018-1487-0 29427197 PMC6003969

[B6] Bentall R. P. (1990). The Illusion of Reality: a Review and Integration of Psychological Research on Hallucinations. *Psychol. Bull.* 107 82–95. 10.1037/0033-2909.107.1.82 2404293

[B7] Bentall R. P. De Sousa P. Varese F. Wickham S. Sitko K. Haarmans M. (2014). From adversity to psychosis: pathways and mechanisms from specific adversities to specific symptoms. *Soc. Psychiatry Psychiatr. Epidemiol.* 49 1011–1022. 10.1007/s00127-014-0914-0 24919446

[B8] Birchwood M. Meaden A. Trower P. Gilbert P. Plaistow J. (2000). The power and omnipotence of voices: subordination and entrapment by voices and significant others. *Psychol. Med.* 30 337–344. 10.1017/S0033291799001828 10824654

[B9] Bleuler E. (1960). *Demencia Precoz. EL grupo de las esquizofrenias [Dementia praecox. The group of schizophrenias].* Buenos Aires: Paidós. 21218204

[B10] Bucci S. Berry K. Barrowclough C. Haddock G. (2016). Family Interventions in Psychosis: a Review of the Evidence and Barriers to Implementation. *Aust. Psychol.* 51 62–68. 10.1111/ap.12172

[B11] Campbell M. L. C. Morrison A. P. (2012). “Subjective experiences of delusion and paranoia,” in *Experiencing Psychosis: Personal and Professional Perspectives*, eds Geekie J. Randal D. Lampshire D. Read J. (East Sussex: Routledge).

[B12] Carvalho S. Pinto-Gouveia J. Pimentel P. Maia D. Gilbert P. Mota-Pereira J. (2013). Entrapment and Defeat Perceptions in Depressive Symptomatology: through an Evolutionary Approach. *Psychiatry* 76 53–67. 10.1521/psyc.2013.76.1.53 23458115

[B13] Cermolacce M. Sass L. Parnas J. (2010). What is bizarre in bizarre delusions? *A critical review*. *Schizophr. Bull.* 36 667–679. 10.1093/schbul/sbq001 20142381 PMC2894592

[B14] Clérambault G. G. (1926). *El Automatismo Mental [Mental Automatism].* Madrid: Dor.

[B15] Corr P. J. (2013). Approach and avoidance behaviour: multiple systems and their interactions. *Emot. Rev.* 5 285–290. 10.1177/1754073913477507

[B16] Deci E. L. Ryan R. M. (2000). The “what” and “why” of goal pursuits: human needs and the self-determination of behavior. *Psychol. Inq.* 11 227–268. 10.1207/S15327965PLI1104_01

[B17] Dudley R. Aynsworth C. Cheetham R. McCarthy-Jones S. Collerton D. (2018). Prevalence and characteristics of multi-modal hallucinations in people with psychosis who experience visual hallucinations. *Psychiatry Res.* 269 25–30. 10.1016/j.psychres.2018.08.032 30145297

[B18] Feyaerts J. Henriksen M. G. Vanheule S. Myin-Germeys I. Sass L. A. (2021). Delusions beyond beliefs: a critical overview of diagnostic, aetiological, and therapeutic schizophrenia research from a clinical-phenomenological perspective. *Lancet Psychiatry* 8 237–249. 33485408 10.1016/S2215-0366(20)30460-0

[B19] Garety P. A. Freeman D. (2013). The past and future of delusions research: from the inexplicable to the treatable. *Br. J. Psychiatry J. Ment. Sci.* 203 327–333. 10.1192/bjp.bp.113.126953 24187067

[B20] Garety P. A. Ward T. Rus-Calafell M. (2020). “Beyond Belief—New Approaches to the Treatment of Paranoia,” in *A Clinical Introduction to Psychosis*, eds Badcock C. J. Paulik G. (Cambridge, Massachusetts: Academic Press), 591–613. 10.1016/B978-0-12-815012-2.00025-0

[B21] Geekie J. Randal P. Lampshire D. Read J. (2012). *Experiencing Psychosis: Personal and Professional Perspectives.* East Sussex, England: Routledge.

[B22] Gilbert P. (2001). Evolutionary approaches to psychopathology: the role of natural defences. *Aust. N. Z. J. Psychiatry* 35 17–27. 10.1046/j.1440-1614.2001.00856.x 11270452

[B23] Gilbert P. McEwan K. Hay J. Irons C. Cheung M. (2007). Social rank and attachment in people with a bipolar disorder. *Clin. Psychol. Psychother.* 14 48–53. 10.1002/cpp.508

[B24] Handest P. Klimpke C. Raballo A. Larøi F. (2016). From Thoughts to Voices: understanding the Development of Auditory Hallucinations in Schizophrenia. *Rev. Philos. Psychol.* 7 595–610. 10.1007/s13164-015-0286-8

[B25] Henriksen M. G. Parnas J. (2017). Clinical Manifestations of Self-disorders in Schizophrenia Spectrum Conditions. *Curr. Probl. Psychiatry* 18 177–183. 10.1515/cpp-2017-0014

[B26] Henriksen M. G. Raballo A. Parnas J. (2016). The Pathogenesis of Auditory Verbal Hallucinations in Schizophrenia: a Clinical–Phenomenological Account. *Philos. Psychiatry Psychol.* 22 165–181. 10.1353/ppp.2015.0041

[B27] Jaspers K. (1913). *Psicopatología general [General Psychopathology].* Buenos Aires: Editorial Beta. 10.53680/vertex.v34i161.489 37819060

[B28] Jones N. Luhrmann T. M. (2016). Beyond the sensory: findings from an in-depth analysis of the phenomenology of “auditory hallucinations” in schizophrenia. *Psychosis* 8 191–202. 10.1080/17522439.2015.1100670

[B29] Kantor J. Smith N. W. (1975). *The science of psychology: An interbehavioral survey.* Chicago, ILL: Principia Press.

[B30] Laframboise H. L. (1973). Health policy?: breaking the problem down into more manageable segments. *Can. Med. Assoc. J.* 108 388–393. 4691098 PMC1941185

[B31] Lakeman R. (2014). The Finnish open dialogue approach to crisis intervention in psychosis: a review. *Psychother. Aust.* 20 28–35.

[B32] Lalonde M. (1974). *A New Perspective on the Health of Canadians.* Ottawa, Ontario, Canada: Information Canada.

[B33] Laroi F. Luhrmann T. M. Bell V. Christian W. A. Deshpande S. Fernyhough C. (2014). Culture and hallucinations: overview and future directions. *Schizophr. Bull.* 40 213–220. 10.1093/schbul/sbu012 24936082 PMC4141319

[B34] Lewin K. (1936). *Principles of topological psychology.* New York: McGraw-Hill.

[B35] Liotti G. Gilbert P. (2011). Mentalizing, motivation, and social mentalities: theoretical considerations and implications for psychotherapy. *Psychol. Psychother. Theory Res. Pract.* 84 9–25. 10.1348/147608310X520094 22903828

[B36] Liotti G. Gumley A. (2009). “An Attachment Perspective on Schizophrenia: The Role of Disorganized Attachment, Dissociation and Mentalization,” in *Psychosis, Trauma and Dissociation: Emerging Perspectives on Severe Psychopathology*, eds Moskowitz A. Schäfer I. Dorahy M. J. (Chichester: John Wiley & Sons), 117–131. 10.1002/9780470699652.ch9

[B37] Lungu A. Linehan M. M. (2017). “Dialectical behavior therapy: Overview, characteristics, and future directions,” in *The Science of Cognitive Behavioral Therapy*, eds Hofmann S. Asmundson G. (New York: Academic Press), 429–459. 10.1016/B978-0-12-803457-6.00018-0

[B38] Maher B. A. (2006). The relationship between delusions and hallucinations. *Curr. Psychiatry Rep.* 8 179–183. 10.1007/s11920-006-0021-3 19817067

[B39] Maijer K. Begemann M. J. H. Palmen S. J. M. C. Leucht S. Sommer I. E. C. (2018). Auditory hallucinations across the lifespan: a systematic review and meta-analysis. *Psychol. Med.* 48 879–888. 10.1017/s0033291717002367 28956518

[B40] Martínez R. Moreno R. (2014). *Cómo Plantear Y Responder Preguntas De Manera Científica [How To Raise And Answer Questions Scientifically].* Madrid: Síntesis.

[B41] Maslow A. H. (1943). A Theory of Human Motivation. *Psychol. Rev.* 50 370–396.

[B42] McCarthy-Jones S. Trauer T. MacKinnon A. Sims E. Thomas N. Copolov D. L. (2014). A new phenomenological survey of auditory hallucinations: evidence for subtypes and implications for theory and practice. *Schizophr. Bull.* 40 225–235. 10.1093/schbul/sbs156 23267192 PMC3885292

[B43] Moritz S. Larøi F. (2008). Differences and similarities in the sensory and cognitive signatures of voice-hearing, intrusions and thoughts. *Schizophr. Res.* 102 96–107. 10.1016/j.schres.2008.04.007 18502102

[B44] Moritz S. Pfuhl G. Lüdtke T. Menon M. Balzan R. P. Andreou C. (2017). A two-stage cognitive theory of the positive symptoms of psychosis. Highlighting the role of lowered decision thresholds. *J. Behav. Ther. Exp. Psychiatry* 56 12–20. 10.1016/j.jbtep.2016.07.004 27501907

[B45] Moritz S. Purdon C. Jelinek L. Chiang B. Hauschildt M. (2018). If it is absurd, then why do you do it? The richer the obsessional experience, the more compelling the compulsion. *Clin. Psychol. Psychother.* 25 210–216. 10.1002/cpp.2155 29154502

[B46] Palazzoli M. S. Boscolo L. Cecchin G. F. Prata G. (1977). Family Rituals A Powerful Tool in Family Therapy. *Fam. Process* 16 445–453. 10.1111/j.1545-5300.1977.00445.x 590473

[B47] Parnas J. Nordgaard J. Vargas S. (2010). The concept of psychosis: a clinical and theoretical analysis. *Clin. Neuropsychiatry* 7 32–37.

[B48] Parnas J. Zandersen M. (2018). Self and schizophrenia: current status and diagnostic implications. *World Psychiatry* 17 221–222. 10.1002/wps.20528 29856572 PMC5980564

[B49] Pienkos E. Giersch A. Hansen M. Humpston C. McCarthy-Jones S. Mishara A. (2019). Hallucinations Beyond Voices: a Conceptual Review of the Phenomenology of Altered Perception in Psychosis. *Schizophr. Bull.* 45 S67–S77. 10.1093/schbul/sby057 30715544 PMC6357976

[B50] Read J. Johnstone L. Taitimu M. (2013). “Models of Madness,” in *Psychological, Social and Biological Approaches to Psychosis*, eds Read J. Dillon J. (Sussex: Routledge), 191–209.

[B51] Reininghaus U. Kempton M. J. Valmaggia L. Craig T. K. J. Garety P. Onyejiaka A. (2016). Stress sensitivity, aberrant salience, and threat anticipation in early psychosis: an experience sampling study. *Schizophr. Bull.* 42 712–722. 10.1093/schbul/sbv190 26834027 PMC4838104

[B52] Ribes E. (2018). *). El estudio científico de la conducta individual: Una introducción a la Teoría de la Psicología. [The scientific study of individual behavior: An introduction to the Theory of Psychology].* México: Manual moderno.

[B53] Rosen C. Chase K. A. Jones N. Grossman L. S. Gin H. Sharma R. P. (2016). Listening to Schneiderian Voices: a Novel Phenomenological Analysis. *Psychopathology* 49 163–171. 10.1159/000446546 27304081 PMC4990463

[B54] Sass L. A. Parnas J. (2003). Schizophrenia. *Consciousness and the Self*. *Schizophr. Bull.* 29 427–444. 10.1093/oxfordjournals.schbul.a007017 14609238

[B55] Séglas J. (1982). “Las alucinaciones verbales en clínica mental [Verbal hallucinations in mental clinic],” in *Delusion in the French Clinic*, eds Colina F. Álvarez J. M. (Madrid: Dor), 167–193.

[B56] Seikkula J. Aaltonen J. Alakare B. Haarakangas K. Keränen J. Lehtinen K. (2006). Five-year experience of first-episode nonaffective psychosis in open-dialogue approach: treatment principles, follow-up outcomes, and two case studies. *Psychother. Res.* 16 214–228. 10.1080/10503300500268490

[B57] Sheaves B. Johns L. Griffith L. Isham L. The McPin Hearing Voices Lived Experience Advisory Panel Kabir T. (2020). Why do patients with psychosis listen to and believe derogatory and threatening voices? 21 reasons given by patients. *Behav. Cogn. Psychother.* 48 631–645. 10.1017/S1352465820000429 32723420

[B58] Strauss J. (2014). Reconceptualizing schizophrenia. *Schizophr. Bull.* 40 97–100. 10.1093/schbul/sbt156 24225692 PMC3934407

[B59] Tully S. Wells A. Pyle M. Hudson J. Gumley A. Kingdon D. (2017). Measuring common responses to psychosis: assessing the psychometric properties of a new measure. *Schizophr. Res.* 181 131–136. 10.1016/j.schres.2016.10.015 27746054

[B60] Van Os J. Reininghaus U. (2016). Psychosis as a transdiagnostic and extended phenotype in the general population. *World Psychiatry* 15 118–124. 10.1002/wps.20310 27265696 PMC4911787

[B61] Varese F. Morrison A. P. Beck R. Heffernan S. Law H. Bentall R. P. (2016). Experiential avoidance and appraisals of voices as predictors of voice-related distress. *Br. J. Clin. Psychol.* 55 320–331. 10.1111/bjc.12102 26752336

[B62] Varese F. Smeets F. Drukker M. Lieverse R. Lataster T. Viechtbauer W. (2012). Childhood adversities increase the risk of psychosis: a meta-analysis of patient-control, prospective-and cross-sectional cohort studies. *Schizophr. Bull.* 38 661–671. 10.1093/schbul/sbs050 22461484 PMC3406538

[B63] Wilkinson S. Bell V. (2016). The Representation of Agents in Auditory Verbal Hallucinations. *Mind Lang.* 31 104–126. 10.1111/mila.12096 26900201 PMC4744949

[B64] Woods A. Jones N. Alderson-Day B. Callard F. Fernyhough C. (2015). Experiences of hearing voices: analysis of a novel phenomenological survey. *Lancet Psychiatry* 2 323–331. 10.1016/S2215-0366(15)00006-1 26360085 PMC4580735

[B65] World Health Organization [WHO]. (2018). *Schizophrenia or other primary psychotic disorders.* Available Online at: https://icd.who.int/browse11/l-m/en#/http%3A%2F%2Fid.who.int%2Ficd%2Fentity%2F405565289 (accessed March 4, 2020).

